# The accuracy of lipid accumulation product to predict metabolic syndrome in PCOS: a meta-analysis and comparative analysis with other indicators

**DOI:** 10.3389/fendo.2026.1709946

**Published:** 2026-04-15

**Authors:** Tingting Liu, Wenjie Bo, Qi Cao, Ruiying Wang, Xinyu Qiao, Yuchan Zhong, Jiagui Liang, Huiqiao Lai, Wei Huang

**Affiliations:** 1 Department of Obstetrics and Gynecology, West China Second University Hospital of Sichuan University, Chengdu, Sichuan, China; 2 Key Laboratory of Birth Defects and Related Diseases of Women and Children of Ministry of Education, Chengdu, Sichuan, China

**Keywords:** diagnostic test accuracy, lipid accumulation product, meta-analysis, metabolic syndrome, pcos

## Abstract

**Background:**

The diagnosis of metabolic syndrome (MetS) in patients with polycystic ovary syndrome (PCOS) is complex. Various indicators are utilized to predict MetS in clinical practice. Nonetheless, there is ongoing debate regarding which indicator possesses a higher predictive value. This study examines the accuracy of the lipid accumulation product (LAP) in screening for MetS among patients with PCOS and compares it with other indicators.

**Methods:**

A systematic literature search was conducted in PubMed, Embase, Web of Science, and the Cochrane Library to identify eligible studies. Outcomes were pooled using the mean difference, odds ratio, and diagnostic accuracy parameters, (including sensitivity, specificity, and the area under the summary receiver operating characteristic (AUROC) curve. Comparative analysis was performed using the Z-test.

**Results:**

A meta-analysis of 11 studies comprising 3720 participants revealed that LAP was significantly elevated in PCOS patients with MetS, with a pooled MD of 2.52 units (P<0.001). LAP demonstrated a strong association with MetS, yielding a pooled OR of 34.31 (P<0.001). For the detection of MetS, LAP exhibited a pooled sensitivity of 87% (95% CI: 79%–92%), specificity of 84% (95% CI: 79%–89%), and an AUROC of 0.92 (95% CI: 0.89–0.94). The AUROC of LAP was significantly superior to that of body mass index, waist circumference, triglyceride levels, and abdominal volume index (P<0.001).

**Conclusion:**

LAP represent as a cost-effective, simple, and a better proxy indicator for screening MetS in the PCOS population.

**Systematic Review Registration:**

https://www.crd.york.ac.uk/PROSPERO/, identifier CRD42025638798.

## Background

Polycystic Ovary Syndrome(PCOS) is a reproductive, metabolic, and psychological disorder that affects the entire life cycle of female patients, affecting 10-13% of women of reproductive age (1). According to the latest clinical practice guidance, the diagnosis of PCOS is based on the presence of menstrual dysfunction or chronic anovulation, clinical or biochemical hyperandrogenism, and polycystic ovarian morphology or elevated anti-Müllerian hormone levels. (1). It is primarily characterized by hyperandrogenism, ovulatory dysfunction, and insulin resistance(IR) (2, 3). PCOS is considered to be a metabolic disorder (4). PCOS involves pathophysiological alterations in glucose, lipid, and protein metabolism, along with abnormalities in endocrine function and ovarian morphology. It is associated not only with infertility, endometrial proliferative lesion, but also increases the risk of metabolic syndrome (MetS) and cardiovascular disorders. (5–7). The most important metabolic features, hyperinsulinemia and IR, affect approximately 50-80% of PCOS patients (8–10). Beyond IR, hyperandrogenism is another major endocrine feature of PCOS that significantly contributes to glucose and metabolic dysregulation. These two factors—IR and hyperandrogenism—interact in a mutually reinforcing manner throughout the disease process, and their interplay exacerbates metabolic disturbances, thereby elevating the risk of MetS and type 2 diabetes mellitus (T2DM). MetS is defined as a cluster of metabolic disorders, including central obesity, impaired glucose tolerance or IR, dyslipidemia, and hypertension (11). Patients with MetS and those with PCOS exhibit some shared clinical symptoms, including obesity, IR, dyslipidemia, and various other metabolic disturbances. The two conditions also overlap in several pathophysiological pathways, including IR, chronic inflammation, and dyslipidemia, which contribute to an increased risk of MetS in individuals with PCOS. Some scholars believe that PCOS is a special manifestation of MetS in the ovaries. The incidence of MetS among patients with PCOS is up to 47.9% (12), markedly higher than the approximately 6.7% prevalence observed in the general population.

Current evidence indicates that MetS adversely affects fertility and pregnancy outcomes (13, 14). Moreover, it has been associated with a 1.5-fold increase in mortality rate and a two-fold elevated risk of cardiovascular and cerebrovascular diseases (15). Furthermore, given its strong association with IR, individuals with MetS faced a high risk of developing T2DM, which is known as a potentially debilitating chronic disease with various macrovascular and microvascular complications, including coronary artery disease, diabetic kidney disease, and stroke (16, 17).

Identifying predictors of MetS in women of reproductive age with PCOS is essential for the prevention and management of infertility and metabolic cardiovascular diseases. However, the current diagnostic criteria for MetS are complex, encompassing three main criteria—the Joint Interim Statement (JIS), the National Cholesterol Education Program Adult Treatment Panel III ((NCEP-ATP III), and the International Diabetes Federation (IDF)—which incorporate components including waist circumference(WC), blood pressure, plasma glucose levels, and triglycerides or high-density lipoprotein cholesterol. This complexity makes early detection of individuals with MetS challenging. Therefore, a simpler, and inexpensive indicator has gained its importance to identify MetS. Currently, the application of tools for the clinical diagnosis of abdominal obesity-related health risks has gained their importance. The anthropometric measurements are simple and cost-effective. The Lipid Accumulation Product(LAP) is a widely used anthropometric index for assessing abdominal obesity in individuals and groups. It only involves two parameters: WC and triglyceride levels. It can reflect body fat distribution and upper body adiposity. Previous studies have demonstrated that LAP shows superior predictive performance for MetS in PCOS patients compared to other anthropometric indices, including WC, waist-to-height ratio(WHtR), body mass index(BMI), visceral adiposity index(VAI) and waist-to-hip ratio(WHR) (18, 19). Nonetheless, a study by Naghshband et al. reported conflicting results, indicating that LAP had lower predictive value than both VAI and triglycerides for identifying MetS (20). Additionally, Shreenidhi et al. also believes that VAI has a higher predictive value than LAP (21). While LAP has been a common focus of earlier studies, there are currently no reviews that have provided conclusive evidence regarding the accuracy of LAP or its suitability for screening MetS. Therefore, we conducted a meta-analysis and review to investigate the relationship between LAP and MetS in PCOS and to explore its clinical implications for diagnosing MetS, as well as to compare the diagnostic value of LAP with other anthropometric methods.

## Methods

The study was conducted in conformity with the most updated Preferred Reporting Items for Systematic Reviews and Meta-Analyses (PRISMA-2020) criteria for reporting (see **Supplementary Table 1**) (22). The detailed protocol of this systematic review was registered with PROSPERO (CRD42025638798) before initiation.

### Search strategy and study selection process

The relevant research conducted computerized searches of four databases: PubMed, Embase, Web of Science, and Cochrane Library, and supplemented the literature data through manual retrieval and other methods of literature tracking. The retrieval keywords included “polycystic ovary syndrome,” “Metabolic Syndrome,” “Lipid Accumulation Product,” and “LAP.” Boolean logic was used to combine the keywords, with “AND” connecting different categories and “OR” within the same category. Both subject terms and free-text words were combined to construct a complete retrieval expression. The retrieval time range extended from the inception of each database to July 9, 2025, without any restrictions on language or publication date. After deduplication, the initial screening was performed based on titles and abstracts; after obtaining the full texts, further evaluation was conducted according to inclusion and exclusion criteria. The initial retrieval and literature screening were independently completed by two researchers to ensure the scientific rigor and reliability of the process. The detailed retrieval strategies and expressions for each database are provided in **Supplementary Table 2**.

### Study selection

#### Inclusion criteria

(1) The study design was observational (cohort, case-control, or crosssectional studies); (2) The study population comprised population with PCOS diagnosed by the 2003 Rotterdam criteria; (3) defined MetS based on any current available diagnostic criteria; (4) The study documented the LAP levels for the MetS and Non-MetS groups separately,; (5) The study investigated the diagnostic accuracy of LAP in predicting MetS.

#### Exclusion criteria

(1) Non-thesis research, including case report, review article, case series, or conference abstract; (2) the full-text was irretrievable; (3) The available data were insufficient, and the authors did not respond to data requests or were unwilling to provide the data.

### Data extraction

Two researchers independently completed the literature screening and data extraction work, strictly adhering to the pre-defined inclusion and exclusion criteria. Initially, irrelevant studies were excluded by reading the titles and abstracts; subsequently, the full texts were reviewed to make the final inclusion decision. The data extraction process followed a unified checklist, collecting information such as the first author, publication year, study region and design type, population characteristics, diagnostic criteria for PCOS or MetS, LAP levels in MetS and non-MetS individuals, the corrected odd ratio (OR) values between LAP and MetS, and the diagnostic performance indicators of LAP (such as Area under the curve, sensitivity, and specificity). If a study used multiple diagnostic criteria for MetS, the preferred order was JIS, NCEP-ATP III, and IDF, with other criteria categorized as “other”.

### Quality assessment

Two reviewers independently evaluated the included studies using the QUADAS-2 tool (23), which encompasses four domains: patient selection, index test, reference standard, and flow and timing. The first three domains are also evaluated for their applicability. Each item is judged as “yes,” “no,” or “unclear.” Risk of bias and applicability are categorized as “low,” “high,” or “unclear.” An overall “low” risk of bias requires all domains to be “low.” A “high” risk is assigned if any domain is rated “high” or if three or more are “unclear.” Similarly, applicability is considered “high” if any domain is “high.” Otherwise, the rating is “moderate”.

### Certainty of evidence assessment

The overall certainty of evidence for the diagnostic performance of LAP in predicting metabolic syndrome in patients with PCOS was evaluated using the Grading of Recommendations Assessment, Development and Evaluation (GRADE) approach. The certainty of evidence was assessed according to five key domains: risk of bias, inconsistency, indirectness, imprecision, and publication bias. As evidence was obtained from observational diagnostic studies, the initial certainty was rated as low, and was further downgraded when serious limitations were identified across the above domains. The final certainty of evidence was classified into four levels: high, moderate, low, and very low.

### Statistical analysis

This study utilized Stata version 18.0 (Stata Corporation, College Station, TX, USA) for the comprehensive analysis of the data. The disparity in LAP levels between MetS and non-MetS subjects was assessed for significance by calculating the mean± standard deviation in both groups, with the standardized mean difference (95% confidence interval, 95%CI) serving as the effect size. Potential incorporation bias (mathematical coupling) should be noted because LAP includes WC and triglycerides, which are also components of the definition of MetS. This overlap may artificially inflate diagnostic accuracy. Data not originally reported in mean and standard deviation format were transformed beforehand (24, 25). Bivariate diagnostic accuracy meta-analyses were conducted to determine the pooled OR value, sensitivity, specificity, and area under the summary receiver operating characteristic (AUROC) curve, along with their respective 95% CIs. An area under 0.5 indicates that LAP lacks the ability to distinguish between subjects with and without MetS. An area of 0.7 to 0.8 is considered to have acceptable diagnostic power, 0.8 to 0.9 is deemed excellent, and an area greater than 0.9 is regarded as outstanding (26). In addition to LAP, the AUROC curves for other indicators were also estimated and then Z-test was used to compare the AUROC curve values between LAP and these indicators. Chi-square tests and Cochrane Q-tests were employed to assess heterogeneity among groups, whereas I^2^ was utilized to quantify the extent of heterogeneity. If I^2^ is less than or equal to 50%, this indicates good homogeneity among trials, which were examined using a fixed-effects model. If I^2^ is greater than 50%, it indicates inadequate homogeneity and elevated heterogeneity, necessitating analysis through a random-effects model. To explore potential sources of heterogeneity, subgroup analyses and meta-regression were performed. Subgroup analyses were conducted according to different MetS diagnostic criteria. Owing to insufficient sample size, further subgroup analyses stratified by region were not performed. Egger’s test was used to identify publication bias in the selected studies. The trim-and-fill method was applied to assess the potential influence of missing studies on the pooled effect size. P-value of<0.05 was considered statistically significant.

## Results

### Selection of studies

The PRISMA flow diagram of the entire study selection process is shown in **Supplementary Figure 1**. A total of 166 articles were retrieved from four databases, with 54 duplicates removed. Of the remaining 112 articles, 68 were excluded based on titles and abstracts due to their irrelevance to LAP, MetS, and PCOS. Subsequently, 44 studies underwent detailed review; however, 4 articles could not be obtained in full text, and the remaining 40 studies were excluded due to insufficient data for the current meta-analysis. Ultimately, 11 studies meeting the inclusion criteria were included in this analysis. (18, 20, 21, 27–34).

### General characteristics of studies and quality assessment

**Figure 1:**
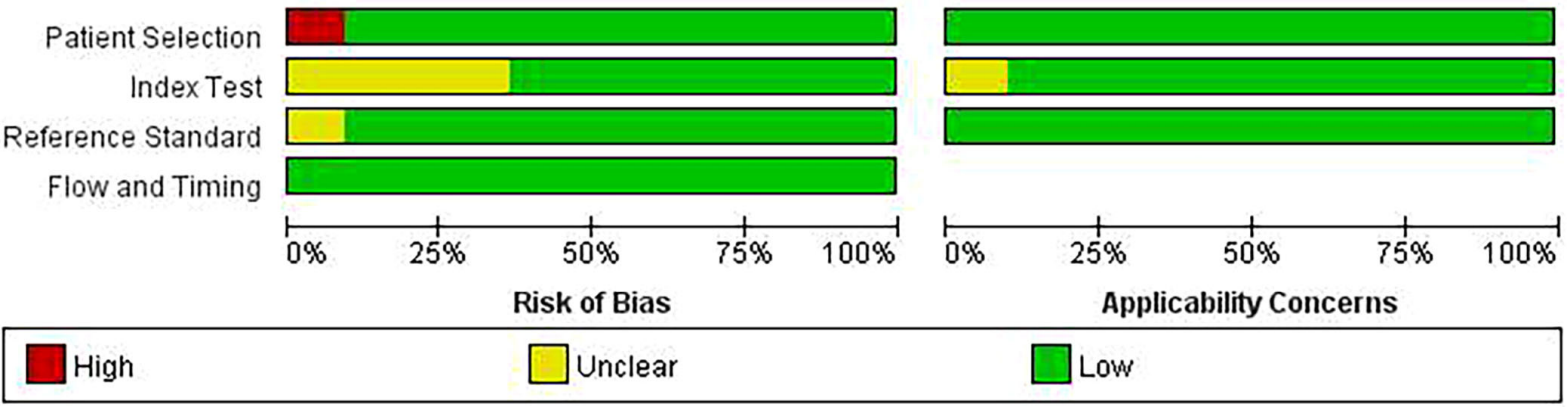
Domain-specific quality assessment results of included studies using the QUADAS-2 tool.

The overall characteristics of the included studies are summarized in **Table 1**. All 11 articles were in English, with 10 being cross-sectional studies and 1 being a case-control study. The MetS group included 779 participants, and the non-MetS group included 2,941 participants, resulting in a total sample size of 3,720. The QUADAS-2 quality assessment revealed that most studies did not explicitly report random or consecutive sampling in the patient selection domain, while the other three domains generally showed low risk. Specifically, the flow and timing domain was consistently rated as low risk (**Figure 1**).

**Table 1:** Characteristics and quality of the included studies.

Author, year	Study location	Study design	PCOS diagnostic criteria	Mets diagnostic criteria	Mets diagnostic criteria	Sample size
					Mets	Non-Mets
Xiang et al., 2013 (34)	China (Asia)	Cross-sectional	the criteria of Rotterdam 2003	IDF	45	60
Macut et al., 2016 (26)	Serbia (Europe)	Cross-sectional	the revised 2003 Rotterdam Consensus conference	NCEP -ATP III,IDF and JIS	36	186
Yin et al., 2021 (6)	China (Asia)	Cross-sectional	Rotterdam criteria	uCDS criterion	124	959
Naghshband et al., 2021 (35)	India (Asia)	Cross-sectional	Rotterdam criteria	IDF	89	61
Babu et al., 2021 (27)	India (Asia)	Cross-sectional	Rotterdam ESHRE/ASRM	NCEP-ATP III	18	78
Yin et al., 2022 (33)	China (Asia)	Cross-sectional	other	other	244	869
Kałuzna et al., 2022 (12)	Poland (Europe)	Cross-sectional	Rotterdam criteria	IDF	64	340
Bir et al., 2023 (28)	India (Asia)	Case-control study	Rotterdam criteria	Other	23	43
Han et al., 2024 (18)	China (Asia)	Cross-sectional	the revised 2003 Rotterdam Consensus conference	NCEP-ATP III	41	106
Shreenidhi, 2024 (1)	India (Asia)	Cross-sectional	Rotterdam criteria	NCEP-ATP III	44	156
Ma et al., 2025 (31)	China (Asia)	Cross-sectional	the Ministry of Public Health of China in 2018	the 2009 International Joint Statement of Multiple Societies	51	83

### Pooled MD meta-analysis

**Figure 2:**
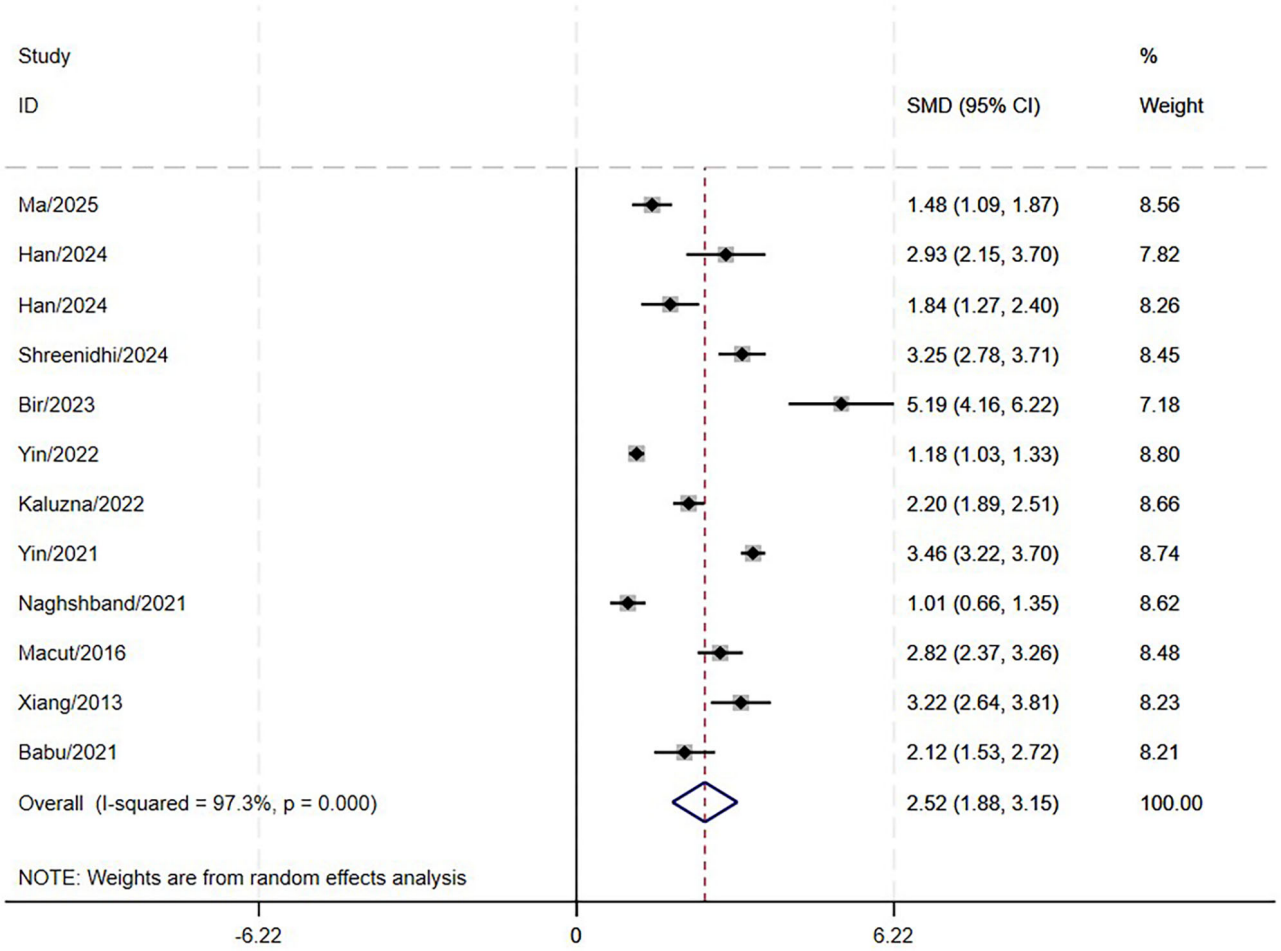
Forest plots of MD meta-analyses of LAP in PCOS with and without MetS.

**Figure 3:**
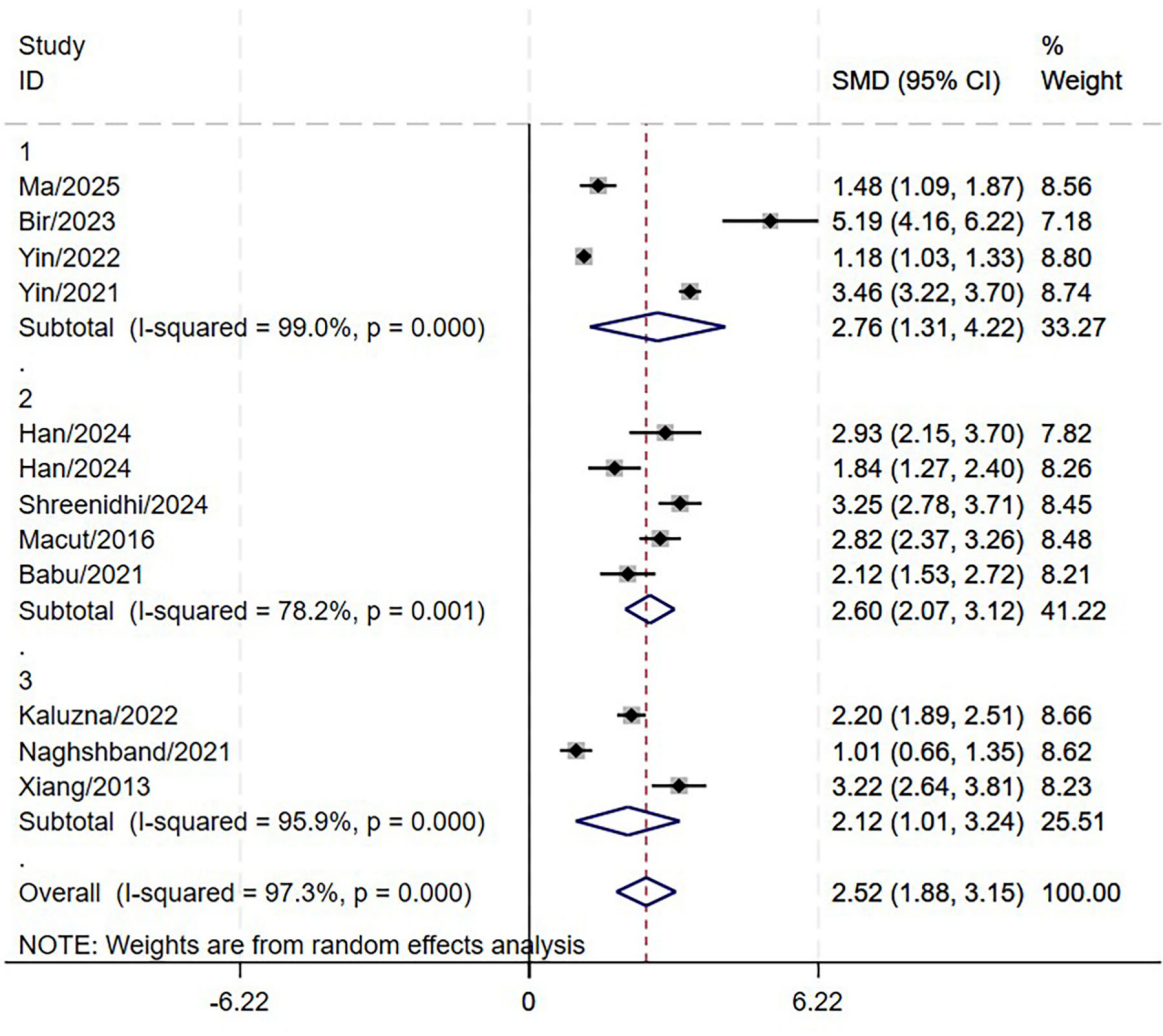
Forest plot of pooled MD by different MetS diagnostic criteria (subgroup analysis).

**Figure 4:**
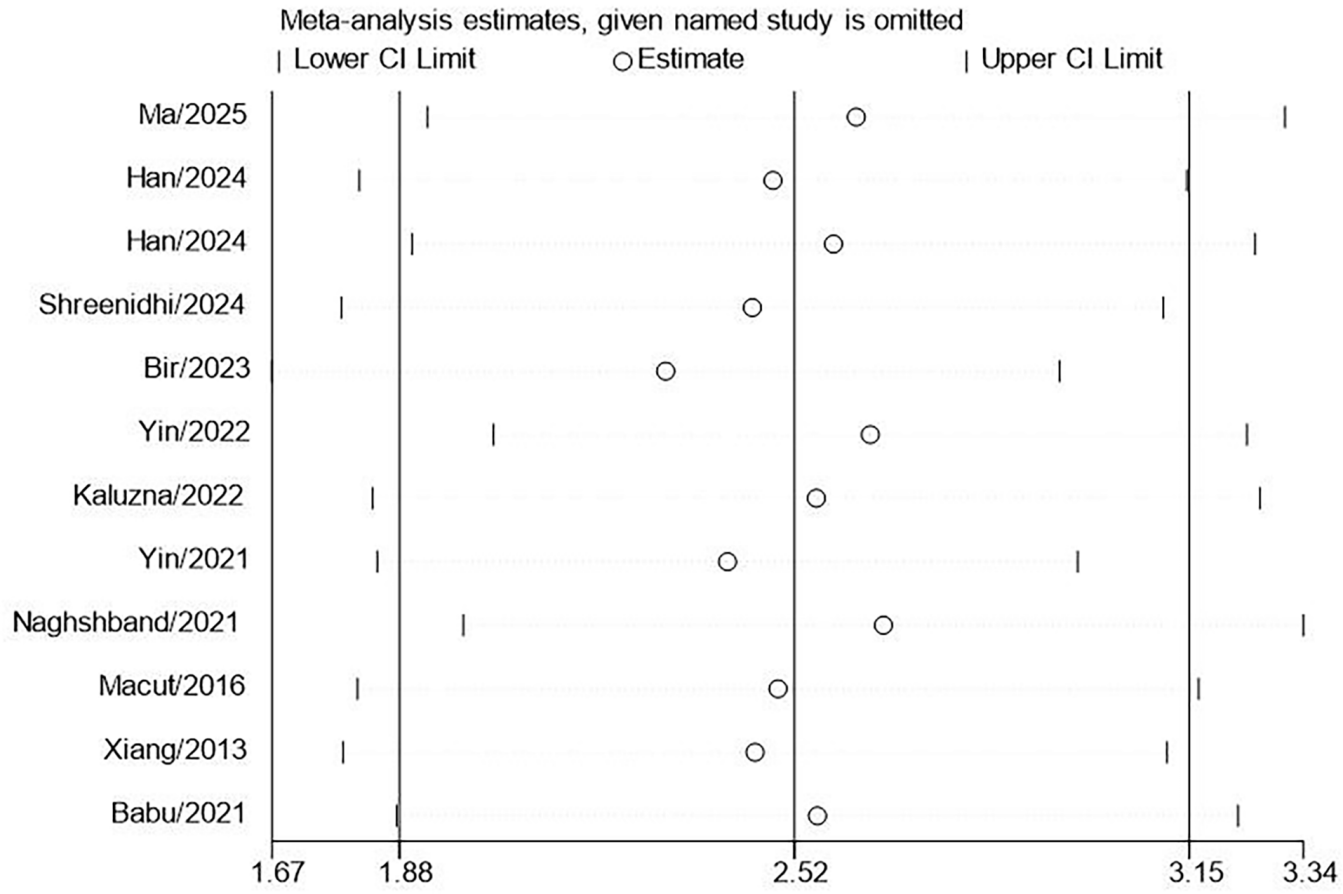
Sensitivity analyses for MD meta-analyses of LAP in PCOS with and without MetS.

This research included 11 studies involving 3,720 patients diagnosed with PCOS to assess the correlation between LAP and MetS. The results indicated that the LAP in PCOS patients with MetS was significantly higher by 2.52 units than those without MetS. (95% CI: 1.88-3.15; P<0.001, **Figure 2**). High heterogeneity was observed across studies (I^2^ = 97.3%). However, substantial heterogeneity persisted in all subgroups stratified by MetS diagnostic criteria (**Figure 3**). Meta-regression analyses revealed no statistically significant associations(coefficient = 0.41, P = 0.474), suggesting that this factor could not fully account for the observed between-study heterogeneity. Sensitivity analysis demonstrated that the aggregated results of this study were reliable (**Figure 4**). The Egger’s test results were insignificant (Z: 1.78; P = 0.106), indicating no potential publication bias. This reveals a notable correlation between an increased LAP and the onset of MetS in individuals with PCOS.

### Pooled OR and diagnostic accuracy meta-analysis

**Figure 5:**
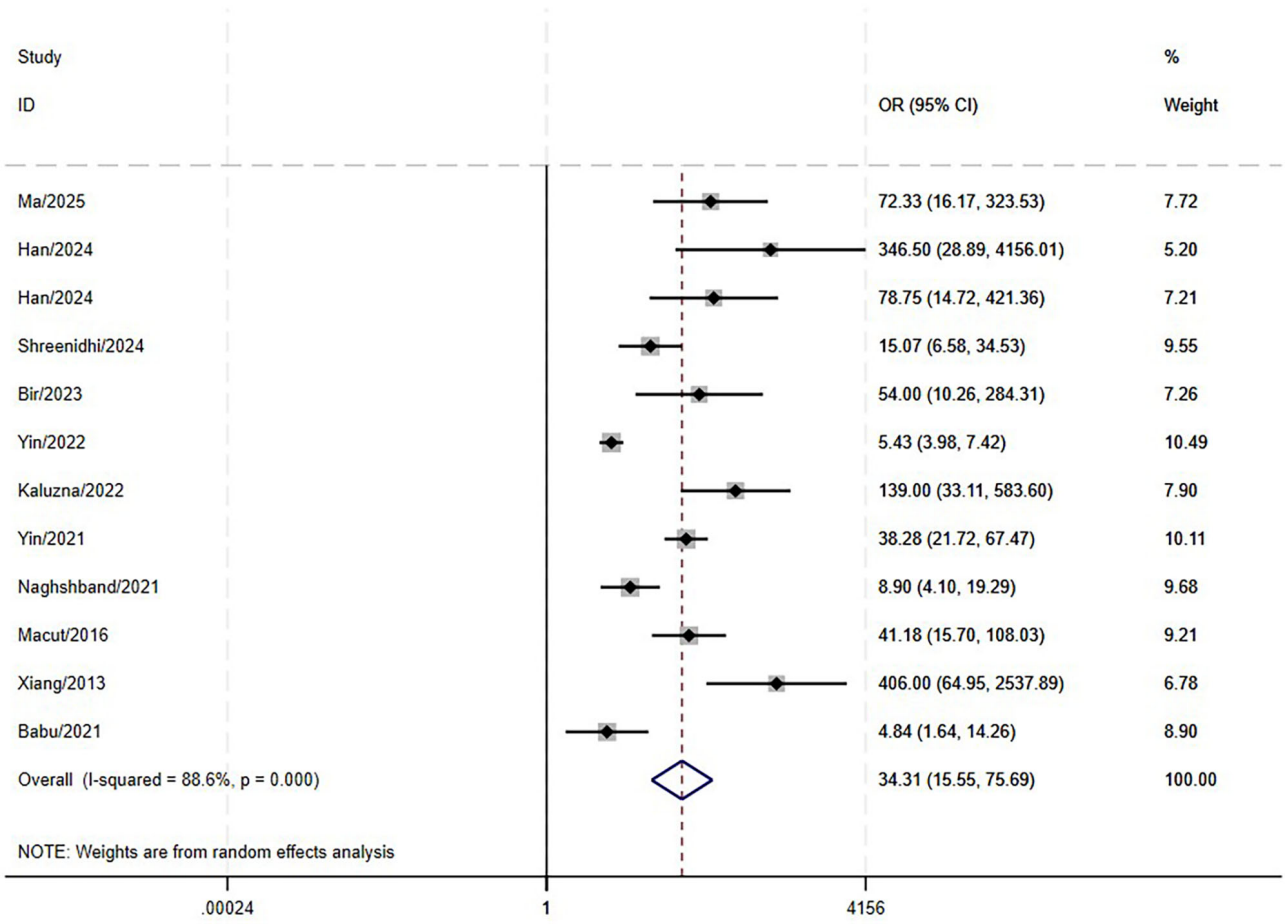
Forest plots of OR meta-analyses between LAP and MetS in PCOS.

**Figure 6:**
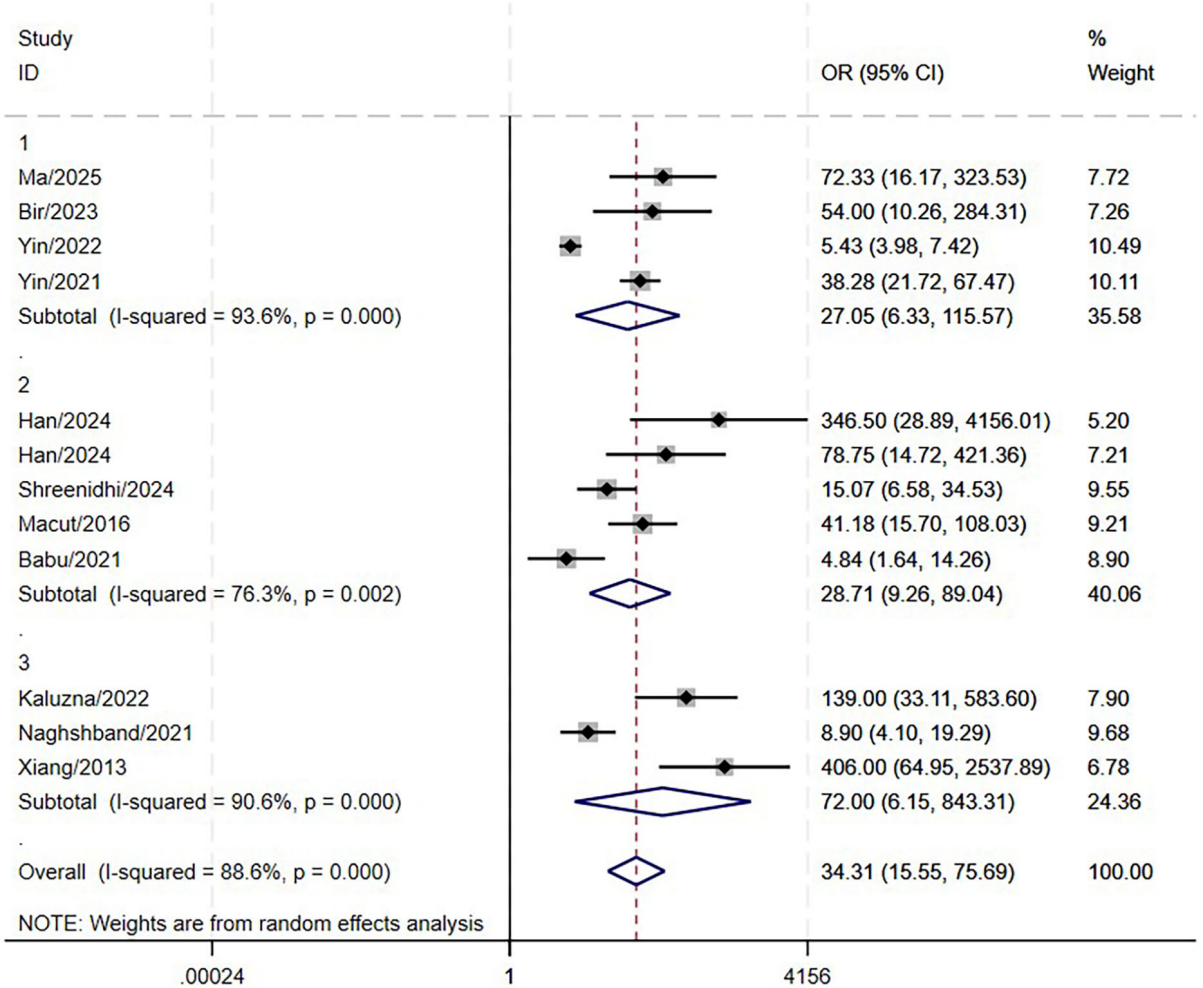
Forest plot of pooled OR by different MetS diagnostic criteria (subgroup analysis).

The results indicated that LAP is significantly associated with MetS.(OR: 34.31; 95% CI: 15.55–75.69; P<0.001, **Figure 5**), with a high level of heterogeneity (I^2^ = 88.6%). Similarly, high heterogeneity persisted in the subgroup analyses (**Figure 6**). No statistically significant association was found in meta-regression analyses (coefficient = -0.29, P = 0.53), implying that this factor did not contribute to the between-study heterogeneity.

**Figure 7:**
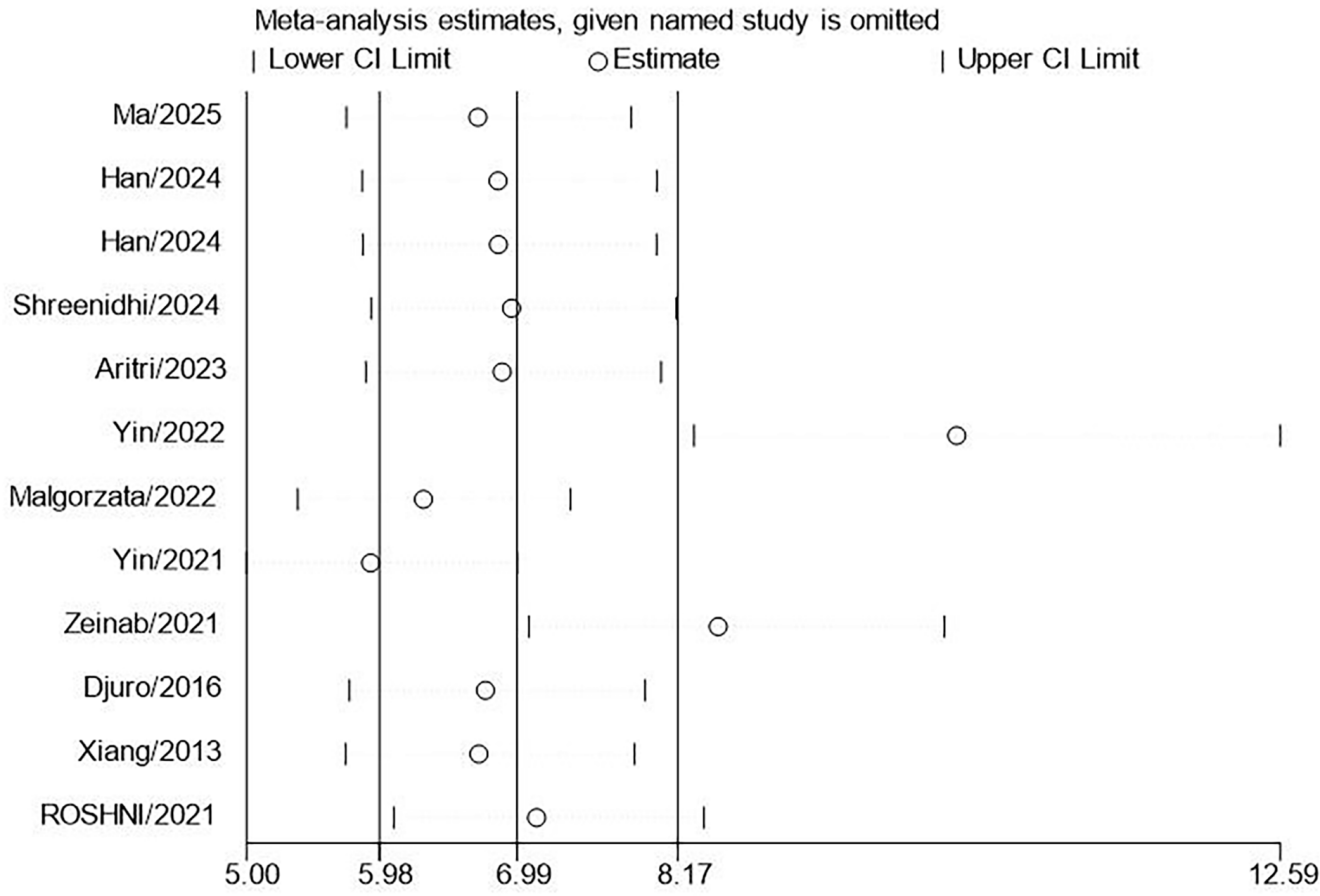
Sensitivity analyses for OR meta-analyses between LAP and MetS in PCOS.

**Figure 8:**
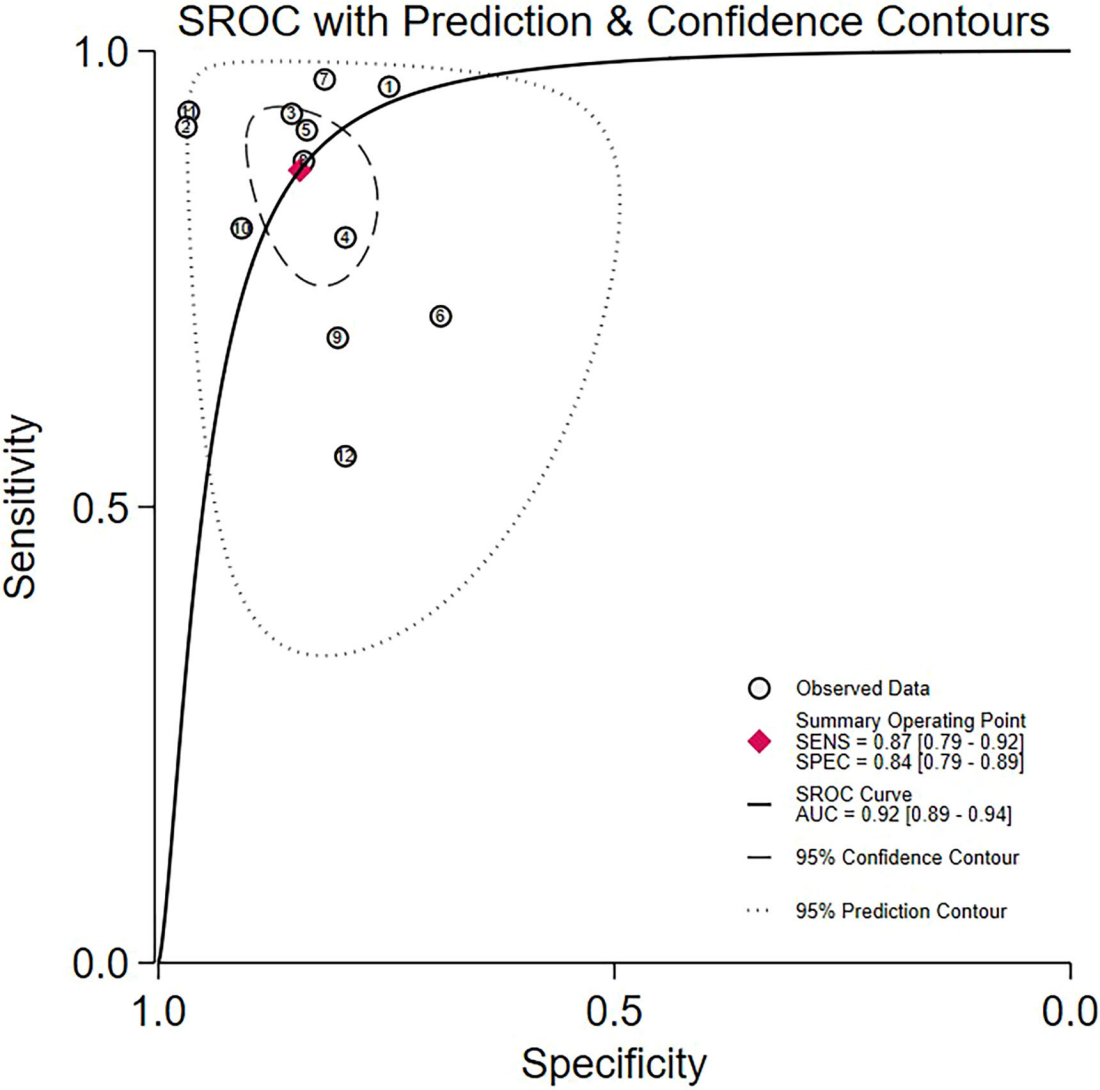
Forest plots of the pooled sensitivity, specificity and AUROC of LAP for the screening of MetS in PCOS.

**Figure 9:**
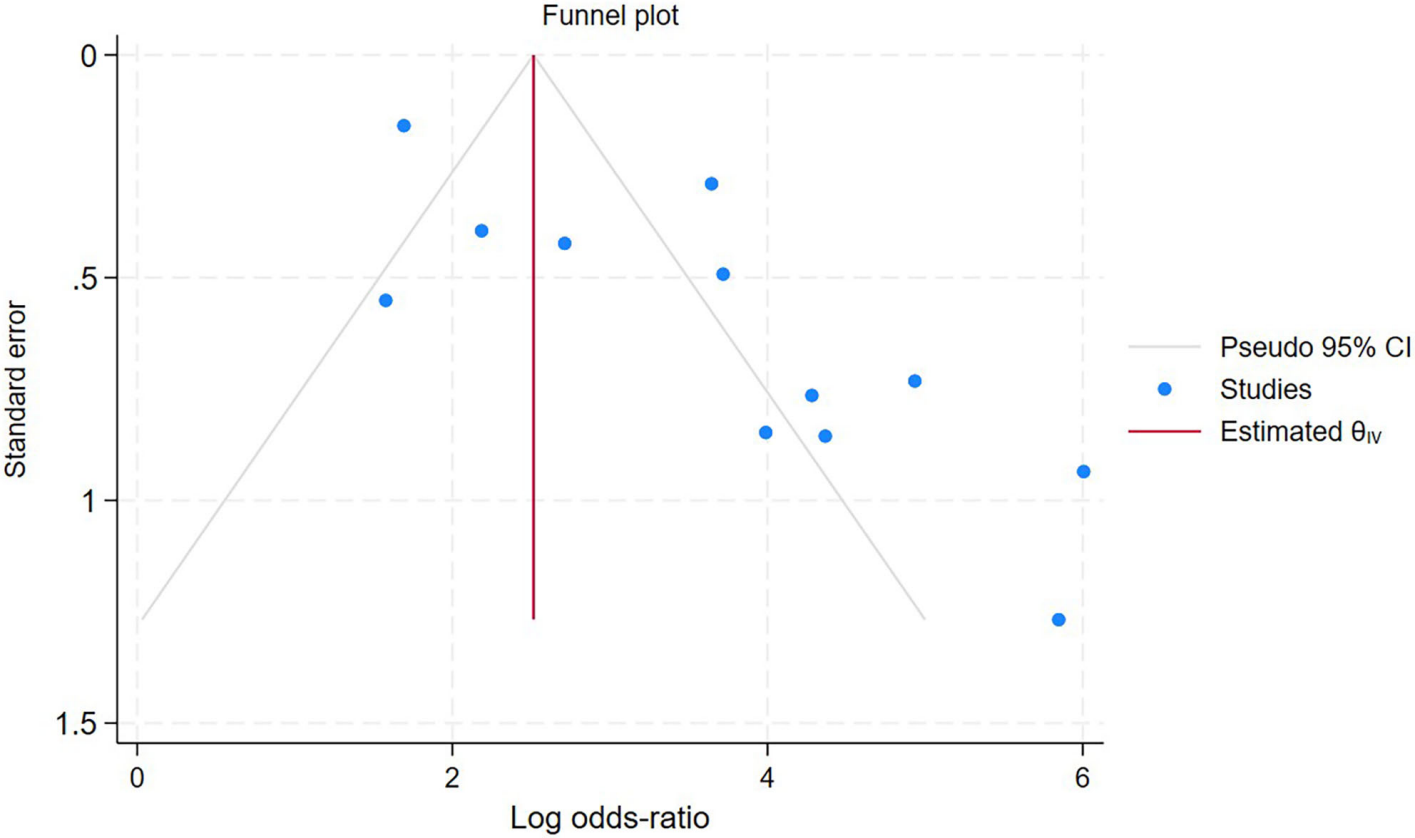
Funnel plots of OR meta-analyses between LAP and MetS in PCOS.

**Figure 10:**
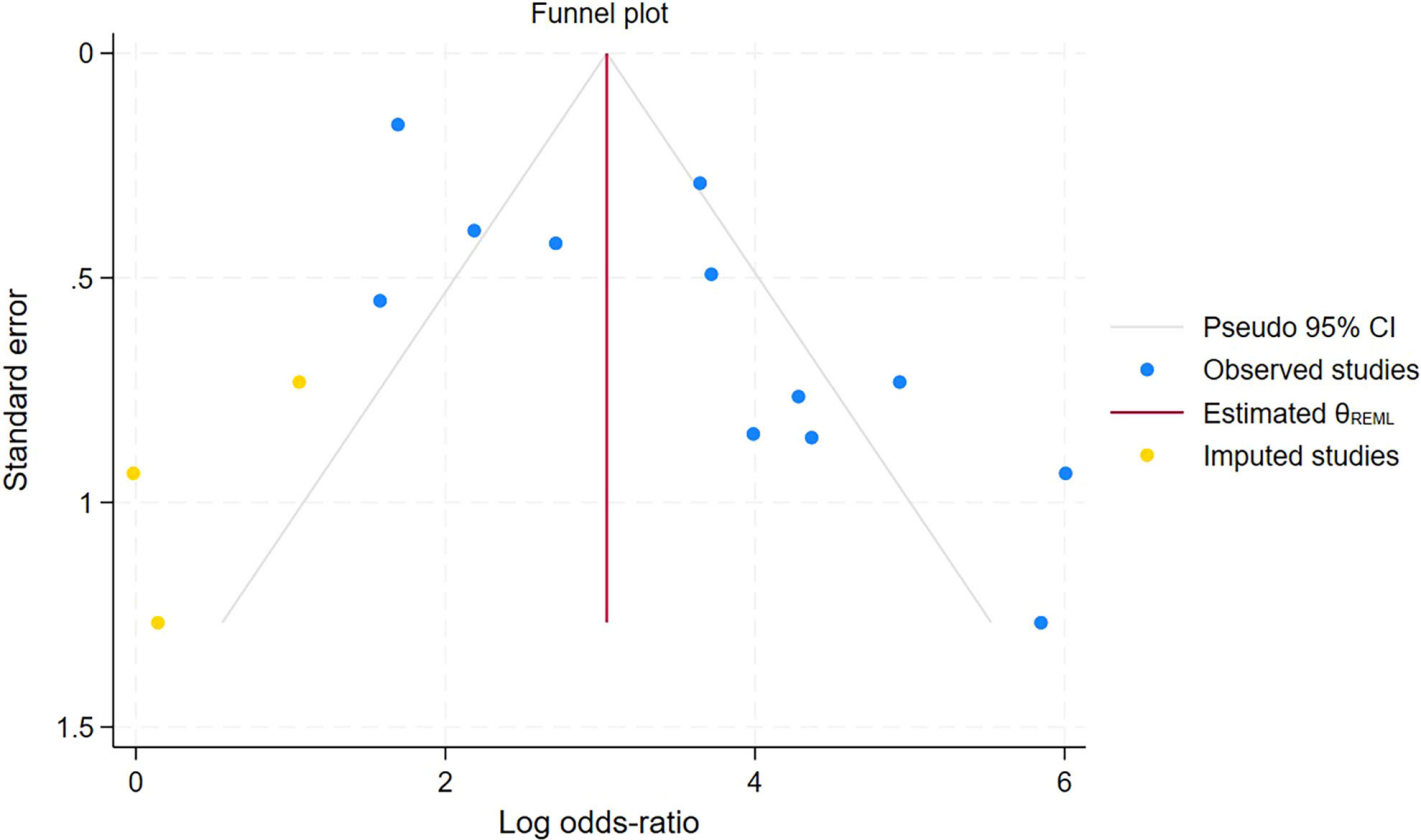
Funnel plots of OR meta-analyses between LAP and MetS in PCOS after utilizing the trim and fill method.

The sensitivity analysis demonstrated that the aggregated results were reliable (**Figure 7**). LAP exhibited a pooled sensitivity of 87% (95% CI: 79%–92%) and a pooled specificity of 84% (95% CI: 79%–89%; **Figure 8**). The AUROC curve analysis revealed a value of 0.92 (95% CI: 0.89–0.94, **Figure 8**), indicating that LAP exhibited excellent screening accuracy for MetS in PCOS. The result of the Deeks’ funnel plot is depicted in **Figure 9**, and the Egger’s test results were significant (Z: 3.69; P = 0.0002), indicating the potential for publication bias. We further utilized the trim and fill method to analyze whether publication bias affected the results. After inputting 3 studies into the funnel plot, the OR value changed from 34.31 (95% CI: 15.43-77.8) to 20.95 (95% CI: 8.69-50.52, **Figure 10**), indicating that the publication bias overestimated the association between LAP and MetS. However, it did not affect the overall result.

### Comparison of diagnostic value between LAP and other indicators

The diagnostic accuracies of indicators, excluding LAP were pooled for comparative analyses. Results showed that the AUROC curves of LAP is significantly higher than other indicators including triglyceride,VAI,BMI and WC. (**Table 2**).

**Table 2:** Direct comparison of the AUROC curves of LAP and other indicators for the screening of MetS.

Indicators	Number of studies	Totle sample	AUROC (95%CI)	Z value	P value
LAP	11	3720	0.92 (0.89-0.94)	ref	ref
Triglyceride	4	1619	0.90 (0.88-0.93)	26.87	<0.001
VAI	5	1993	0.89 (0.86-0.92)	38.11	<0.001
BMI	4	1889	0.88 (0.85-0.91)	49.83	<0.001
WC	4	1485	0.83 (0.80-0.86)	102.30	<0.001

### Certainty of evidence

The GRADE framework was used to assess evidence certainty, and the overall certainty was rated as low. The main reasons included substantial between-study heterogeneity and potential publication bias, which led to the downgrading of evidence quality. The detailed GRADE evidence profile is presented in **Table 3**.

**Table 3:** GRADE Summary of findings for the diagnostic performance of LAP in predicting MetS in PCOS.

Outcome	No. of studies	Participants	Risk of bias	Inconsistency	Indirectness	Imprecision	Publication bias	Effect estimate (95% CI)	Certainty of evidence (GRADE)
Sensitivity	11	3720	Not serious	Serious	Not serious	Not serious	Serious	0.87 (0.79–0.92)	Low ⨁⨁◯◯
Specificity	11	3720	Not serious	Serious	Not serious	Not serious	Serious	0.84 (0.79–0.89)	Low ⨁⨁◯◯
AUROC	11	3720	Not serious	Serious	Not serious	Not serious	Serious	0.92 (0.89–0.94)	Low ⨁⨁◯◯

Evidence was downgraded one level for inconsistency due to substantial heterogeneity across studies (I^2^ > 50%). Evidence was downgraded one level for publication bias based on significant Egger’s test results. LAP, lipid accumulation product; VAI, visceral adiposity index; BMI, body mass index; WC, waist circumference; AUROC, area under the summary receiver operating characteristic; MetS, metabolic syndrome; PCOS, polycystic ovary syndrome; LAP, lipid accumulation product.

## Discussion

### Main findings

In this systematic review and meta-analysis, 11 studies with 3720 participants from various countries were included. The current meta-analysis demonstrated that patients with PCOS and MetS had significantly higher mean LAP values than those without MetS. LAP was also positively associated with MetS, suggesting that an increase in LAP would increase the risk of MetS in PCOS patients. As a screening tool for MetS, LAP exhibited high sensitivities and specificities, with AUROC curves of 0.92, indicating that showed favorable diagnostic performance for MetS in patients with PCOS. Moreover, LAP had a superior diagnostic ability for MetS compared to triglyceride, VAI, BMI and WC.

PCOS is an endocrine disorder marked by IR, hyperinsulinemia, obesity, dyslipidemia, and various metabolic alterations. The pathogenesis of PCOS is intricate, and current findings indicates a strong association between PCOS and MetS. Several studies have proved that MetS in PCOS is associated with certain metabolic disorders, including fatty liver disease (36), atherosclerosis, and cerebrovascular disease witch cause significant harm to physical health. However, there is no unified diagnostic criteria for metabolic syndrome; the criteria include those set by the IDF, NCEP-ATP III, and JIS. All these criteria, which involve obesity-related indicators, blood pressure, plasma glucose levels, and triglycerides or high-density lipoprotein cholesterol, are complicated to achieve in clinical practice. Several simple indicators are used to identify MetS, including those developed solely from anthropometric measurements, such as WC, weight, and height. Triglycerides are derived from biochemical measurements alone, while LAP and VAI combine anthropometric measurements with biochemical markers. However, there is no consensus on which indicator offers superior predictive value.

The meta-analysis revealed that patients with PCOS and MetS exhibited significantly higher mean LAP values (2.52 units) compared to those without MetS, with no evidence of publication bias and a high level of heterogeneity (I^2^=97.3%). Subgroup analyses did not fully eliminate the significant heterogeneity across included studies, suggesting that the variability in diagnostic performance may be affected by multiple complex factors. Consequently, the pooled diagnostic estimates should be interpreted cautiously. LAP was initially tested in 2005 as a more effective tool than BMI for detecting cardiovascular risks in adults. It combined WC and triglycerides levels to describe anatomical and physiological changes (29). []These two parameters collectively characterize the body’s abnormal fat storage capacity. An elevated level of LAP essentially reveals ectopic lipid deposition, where fat is pathologically accumulated in non-adipose tissues such as the liver, skeletal muscle, heart, blood vessels, kidneys, and pancreas (29, 37). Notably, this pathological process of visceral fat accumulation is closely related to a vicious cycle between PCOS and MetS (11). Specifically, visceral fat accumulation promotes ectopic lipid deposition, exacerbating IR. Meanwhile, the hyperandrogenism typical of PCOS further impairs the normal function of adipose tissue, creating a self-reinforcing cycle of metabolic disorder. This bidirectional pathological-physiological link not only explains the high incidence of MetS in PCOS but also highlights the unique value of LAP as an assessment tool for MetS (38).

The meta-analysis conducted by Bendix et al. in 2025 revealed that community residents with MetS had significantly higher mean LAP values compared to those without MetS. Additionally, LAP exhibited moderate-to-high AUROC value of 0.88 in the detection of MetS, a finding that aligns with our results in patients with PCOS (39). Furthermore, our findings reveal that LAP had a higher AUROC value of 0.92 in predicting MetS in patients with PCOS, indicating that LAP possesses greater diagnostic value in this patient group compared to the general population. This may be attributed to the fact that patients with PCOS and metabolic syndrome tend to have higher LAP values than those in the general population with metabolic syndrome.

Furthermore, we concluded that the diagnostic value of LAP is significantly higher than that of BMI, WC, triglycerides, and VAI. The remaining indicators were not included in the comparison due to the small sample size (number of studies less than 4). While BMI is a standard tool for evaluating obesity, its accuracy is limited as it only provides a general approximation of body fat (35). The precise localization of fat is considered crucial due to its distinct metabolic implications (40). WC is considered a direct estimate of abdominal obesity. Studies have confirmed that waist circumference is defective in predicting MetS due to the influence of height (41, 42). Some studies have indicated that the LAP index and VAI are superior predictors compared to anthropometric parameters, as they integrate anatomical and physiological changes associated with fat accumulation (19, 43). A meta-analysis conducted by 44 found that VAI had a higher AUROC curve of 0.847 for detecting MetS in the general population (44). Another meta-analysis concluded that LAP (0.88) and VAI (0.85) had comparable abilities to predict MetS in the general female population (39). When qualitatively compared with our study’s findings, LAP can be regarded as a better proxy indicator than VAI for application in daily clinical practice. LAP is derived from only two parameters-WC and triglycerides, whereas VAI relies on a more complex formula involving multiple variables including WC, triglycerides, BMI, and high density lipoprotein cholesterol (14, 45). This evidence further supports the use of LAP as an efficient and practical marker, reducing the cumulative cost in MetS screening in large PCOS populations (46).

### Strengths and limitations

This study represents the first systematic review and meta-analysis to investigate LAP as a potential screening tool for MetS in PCOS and to compare it with other indicators. Yet, some limitations still exist. First, the small number of included studies and limited number of MetS cases. Second, despite performing subgroup analyses and meta-regression to explore potential sources of heterogeneity, considerable heterogeneity persisted and remained unexplained. Third, significant publication bias was detected in this meta-analysis, suggesting that unpublished studies with negative results may be missing. Although the overall result remained unchanged after employing the trim and fill method to input 3 studies into the funnel plot, the presence of strong publication bias may limit the confidence and generalizability of the pooled effect size. Fourthly, although this study statistically compared the diagnostic performance of LAP with other indicators, the data for these comparisons were derived exclusively from studies included in the current meta-analysis and some indicators were not included duo to the insufficient number of studies(n < 4). Furthermore, a major limitation of this study is the presence of incorporation bias due to mathematical coupling. LAP includes WC and triglyceride, which are also used to define MetS. All the above limitations may result in overestimation of diagnostic accuracy, and the findings should therefore be interpreted cautiously.

## Conclusion

This meta-analysis indicates that LAP serves as a superior proxy indicator for predicting MetS in women with PCOS, and performs better than other comparable indices. Considering the increasing prevalence of MetS among individuals with PCOS and the significant economic burden associated with MetS, the results of this study could support the use of LAP in a large scale population screenings, particularly in settings with limited resources. However, significant heterogeneity exists among the studies; future multi-center prospective studies are needed to further evaluate the predictive value of LAP in different ethnicities, age groups, and MetS diagnostic criteria. Additionally, large-scale prospective cohort studies are required to validate its clinical applicability.

## Data Availability

The original contributions presented in the study are included in the article/**Supplementary Material**. Further inquiries can be directed to the corresponding author.
