# Supplementary material for: The accuracy of lipid accumulation product to predict metabolic syndrome in PCOS: a meta-analysis and comparative analysis with other indicators
Source: Front Endocrinol (Lausanne). 2026 Apr 15;17:1709946. doi: 10.3389/fendo.2026.1709946 (PMC13124632; doi:10.3389/fendo.2026.1709946)
Supplement: Supplementary file 1 [file DataSheet1.pdf]

Supplementary Material

Supplementary table 1. The most updated Preferred Reporting Items for Systematic Reviews and Meta-Analyses (PRISMA-2020) criteria

Section and Topic	Item #	Checklist item	Location where item is reported
**TITLE**			
Title	1	Identify the report as a systematic review.	Title
**ABSTRACT**			
Abstract	2	See the PRISMA 2020 for Abstracts checklist.	Abstract
**INTRODUCTION**			
Rationale	3	Describe the rationale for the review in the context of existing knowledge.	Background
Objectives	4	Provide an explicit statement of the objective(s) or question(s) the review addresses.	Background
**METHODS**			
Eligibility criteria	5	Specify the inclusion and exclusion criteria for the review and how studies were grouped for the syntheses.	Methods
Information sources	6	Specify all databases, registers, websites, organisations, reference lists and other sources searched or consulted to identify studies. Specify the date when each source was last searched or consulted.	Methods
Search strategy	7	Present the full search strategies for all databases, registers and websites, including any filters and limits used.	Methods
Selection process	8	Specify the methods used to decide whether a study met the inclusion criteria of the review, including how many reviewers screened each record and each report retrieved, whether they worked independently, and if applicable, details of automation tools used in the process.	NA
Data collection process	9	Specify the methods used to collect data from reports, including how many reviewers collected data from each report, whether they worked independently, any processes for obtaining or confirming data from study investigators, and if applicable, details of automation tools used in the process.	Methods
Data items	10a	List and define all outcomes for which data were sought. Specify whether all results that were compatible with each outcome domain in each study were sought (e.g. for all measures, time points, analyses), and if not, the methods used to decide which results to collect.	Methods
	10b	List and define all other variables for which data were sought (e.g. participant and intervention characteristics, funding sources). Describe any assumptions made about any missing or unclear information.	Methods
Study risk of bias assessment	11	Specify the methods used to assess risk of bias in the included studies, including details of the tool(s) used, how many reviewers	Methods

Section and Topic	Item #	Checklist item	Location where item is reported
		assessed each study and whether they worked independently, and if applicable, details of automation tools used in the process.	
Effect measures	12	Specify for each outcome the effect measure(s) (e.g. risk ratio, mean difference) used in the synthesis or presentation of results.	Methods
Synthesis methods	13a	Describe the processes used to decide which studies were eligible for each synthesis (e.g. tabulating the study intervention characteristics and comparing against the planned groups for each synthesis (item #5)).	NA
	13b	Describe any methods required to prepare the data for presentation or synthesis, such as handling of missing summary statistics, or data conversions.	Methods
	13c	Describe any methods used to tabulate or visually display results of individual studies and syntheses.	NA
	13d	Describe any methods used to synthesize results and provide a rationale for the choice(s). If meta-analysis was performed, describe the model(s), method(s) to identify the presence and extent of statistical heterogeneity, and software package(s) used.	Methods
	13e	Describe any methods used to explore possible causes of heterogeneity among study results (e.g. subgroup analysis, meta-regression).	Methods
	13f	Describe any sensitivity analyses conducted to assess robustness of the synthesized results.	Methods
Reporting bias assessment	14	Describe any methods used to assess risk of bias due to missing results in a synthesis (arising from reporting biases).	NA
Certainty assessment	15	Describe any methods used to assess certainty (or confidence) in the body of evidence for an outcome.	NA
**RESULTS**			
Study selection	16a	Describe the results of the search and selection process, from the number of records identified in the search to the number of studies included in the review, ideally using a flow diagram.	Results
	16b	Cite studies that might appear to meet the inclusion criteria, but which were excluded, and explain why they were excluded.	Results
Study characteristics	17	Cite each included study and present its characteristics.	Results
Risk of bias in studies	18	Present assessments of risk of bias for each included study.	Results
Results of individual studies	19	For all outcomes, present, for each study: (a) summary statistics for each group (where appropriate) and (b) an effect estimate and its precision (e.g. confidence/credible interval), ideally using structured tables or plots.	Results
Results of syntheses	20a	For each synthesis, briefly summarise the characteristics and risk of bias among contributing studies.	Results
	20b	Present results of all statistical syntheses conducted. If meta-analysis was done, present for each the summary estimate and its precision (e.g. confidence/credible interval) and measures of statistical heterogeneity. If comparing groups, describe the direction of the effect.	Results
	20c	Present results of all investigations of possible causes of	NA

Section and Topic	Item #	Checklist item	Location where item is reported
		heterogeneity among study results.	
	20d	Present results of all sensitivity analyses conducted to assess the robustness of the synthesized results.	Results
Reporting biases	21	Present assessments of risk of bias due to missing results (arising from reporting biases) for each synthesis assessed.	Results
Certainty of evidence	22	Present assessments of certainty (or confidence) in the body of evidence for each outcome assessed.	NA
**DISCUSSION**			
Discussion	23a	Provide a general interpretation of the results in the context of other evidence.	Discussion
	23b	Discuss any limitations of the evidence included in the review.	Discussion
	23c	Discuss any limitations of the review processes used.	Discussion
	23d	Discuss implications of the results for practice, policy, and future research.	Discussion
**OTHER INFORMATION**			
Registration and protocol	24a	Provide registration information for the review, including register name and registration number, or state that the review was not registered.	Methods
	24b	Indicate where the review protocol can be accessed, or state that a protocol was not prepared.	Methods
	24c	Describe and explain any amendments to information provided at registration or in the protocol.	NA
Support	25	Describe sources of financial or non-financial support for the review, and the role of the funders or sponsors in the review.	Funding
Competing interests	26	Declare any competing interests of review authors.	Conflict of Interest
Availability of data, code and other materials	27	Report which of the following are publicly available and where they can be found: template data collection forms; data extracted from included studies; data used for all analyses; analytic code; any other materials used in the review.	NA

Supplementary table 2. The detailed retrieval strategies and expressions for each database.

The retrieval time: 20250709

Sup Table 1 PubMed (n =24)

Search	Query
#1	"polycystic ovary syndrome"[MeSH Terms] OR "Ovary Syndrome, Polycystic"[Title/Abstract] OR "Syndrome, Polycystic Ovary"[Title/Abstract] OR "Polycystic Ovarian Syndrome"[Title/Abstract] OR "Ovarian Syndrome, Polycystic"[Title/Abstract] OR "Polycystic Ovary Syndrome 1"[Title/Abstract] OR

Search	Query
	"Sclerocystic Ovarian Degeneration"[Title/Abstract] OR "Ovarian Degeneration, Sclerocystic"[Title/Abstract] OR "Sclerocystic Ovary Syndrome"[Title/Abstract] OR "Stein-Leventhal Syndrome"[Title/Abstract] OR "Stein Leventhal Syndrome"[Title/Abstract] OR "Syndrome, Stein-Leventhal"[All Fields] OR "Sclerocystic Ovaries"[Title/Abstract] OR "Ovary, Sclerocystic"[Title/Abstract] OR "Sclerocystic Ovary"[Title/Abstract] OR PCOS[Title/Abstract]
#2	((((((((((((((((((((((Metabolic Syndrome[MeSH Terms]) OR (Metabolic Syndromes[Title/Abstract])) OR (Syndrome, Metabolic[Title/Abstract])) OR (Syndromes, Metabolic[Title/Abstract])) OR (Reaven Syndrome X[Title/Abstract])) OR (Syndrome X, Reaven[Title/Abstract])) OR (Metabolic Syndrome X[Title/Abstract])) OR (Insulin Resistance Syndrome X[Title/Abstract])) OR (Metabolic Cardiovascular Syndrome[Title/Abstract])) OR (Cardiovascular Syndrome, Metabolic[Title/Abstract])) OR (Cardiovascular Syndromes, Metabolic[Title/Abstract])) OR (Syndrome, Metabolic Cardiovascular[Title/Abstract])) OR (Syndrome X, Insulin Resistance[Title/Abstract])) OR (Metabolic X Syndrome[Title/Abstract])) OR (Syndrome, Metabolic X[Title/Abstract])) OR (X Syndrome, Metabolic[Title/Abstract])) OR (Syndrome X, Metabolic[Title/Abstract])) OR (Dysmetabolic Syndrome X[Title/Abstract])) OR (Syndrome X, Dysmetabolic[Title/Abstract])) OR (Cardiometabolic Syndrome[Title/Abstract])) OR (Cardiometabolic Syndromes[Title/Abstract])) OR (Syndrome, Cardiometabolic[Title/Abstract])) OR (Syndromes, Cardiometabolic[Title/Abstract])
#3	(((((Lipid Accumulation Product[MeSH Terms]) OR (Accumulation Product, Lipid[Title/Abstract])) OR (Accumulation Products, Lipid[Title/Abstract])) OR (Lipid Accumulation Products[Title/Abstract])) OR (Product, Lipid Accumulation[Title/Abstract])) OR (Products, Lipid Accumulation[Title/Abstract])
#4	#1 AND #2 AND #3

Sup Table 2 Embase (n = 43)

Search	Query
#1	('ovary polycystic disease'/exp OR 'cystic ovary':ab,ti OR 'micropolycystic ovary':ab,ti OR 'multiple follicle cyst':ab,ti OR 'ovary polycystic syndrome':ab,ti OR 'ovary, micropolycystic':ab,ti OR 'ovary, polycystic':ab,ti OR 'polycystic ovarian disease':ab,ti OR 'polycystic ovary':ab,ti OR 'polycystic ovary disease':ab,ti OR 'polycystic ovary syndrome':ab,ti OR 'stein cohen leventhal syndrome':ab,ti OR 'stein leventhal disease':ab,ti OR 'stein leventhal syndrome':ab,ti OR 'syndrome stein leventhal':ab,ti OR 'ovary polycystic disease':ab,ti）
#2	（'metabolic syndrome x'/exp OR 'insulin resistance syndrome':ab,ti OR 'metabolic syndrome':ab,ti OR 'metabolic x syndrome':ab,ti OR (metsyn:ab,ti AND 'metabolic syndrome':ab,ti) OR 'reaven syndrome':ab,ti OR 'reaven syndrome x':ab,ti OR 'reaven`s

Search	Query
	syndrome':ab,ti OR 'reaven`s syndrome x':ab,ti OR 'syndrome of insulin resistance':ab,ti OR 'syndrome x, metabolic':ab,ti OR 'metabolic syndrome x':ab,ti）
#3	（'lipid accumulation product index'/exp OR ('lap index':ab,ti AND lipid:ab,ti) OR 'lipid accumulation product':ab,ti OR 'lipid accumulation product index':ab,ti）
#4	#1 AND #2 AND #3

Sup Table 3 Web of Science (n = 94)

Search	Query
#1	((((((((((((TS=(polycystic ovary syndrome)) OR TS=(Ovary Syndrome, Polycystic)) OR TS=(Syndrome, Polycystic Ovary)) OR TS=(Polycystic Ovarian Syndrome)) OR TS=(Ovarian Syndrome, Polycystic)) OR TS=(Polycystic Ovary Syndrome 1)) OR TS=(Sclerocystic Ovarian Degeneration)) OR TS=(Ovarian Degeneration, Sclerocystic)) OR TS=(Sclerocystic Ovary Syndrome)) OR TS=(Stein-Leventhal Syndrome)) OR TS=(Stein Leventhal Syndrome)) OR TS=(Syndrome, Stein-Leventhal)) OR TS=(Sclerocystic Ovaries)) OR TS=(Ovary, Sclerocystic)) OR TS=(Sclerocystic Ovary)
#2	((((((((((((((((((((TS=(Metabolic Syndrome)) OR TS=(Metabolic Syndromes)) OR TS=(Syndrome, Metabolic)) OR TS=(Syndromes, Metabolic)) OR TS=(Reaven Syndrome X)) OR TS=(Syndrome X, Reaven)) OR TS=(Metabolic Syndrome X)) OR TS=(Insulin Resistance Syndrome X)) OR TS=(Metabolic Cardiovascular Syndrome)) OR TS=(Cardiovascular Syndrome, Metabolic)) OR TS=(Cardiovascular Syndromes, Metabolic)) OR TS=(Syndrome, Metabolic Cardiovascular)) OR TS=(Syndrome X, Insulin Resistance)) OR TS=(Metabolic X Syndrome)) OR TS=(Syndrome, Metabolic X)) OR TS=(X Syndrome, Metabolic)) OR TS=(Syndrome X, Metabolic)) OR TS=(Dysmetabolic Syndrome X)) OR TS=(Syndrome X, Dysmetabolic)) OR TS=(Cardiometabolic Syndrome)) OR TS=(Cardiometabolic Syndromes)) OR TS=(Syndrome, Cardiometabolic)) OR TS=(Syndromes, Cardiometabolic)
#3	(((((TS=(Lipid Accumulation Product)) OR TS=(Accumulation Product, Lipid)) OR TS=(Accumulation Products, Lipid)) OR TS=(Lipid Accumulation Products)) OR TS=(Product, Lipid Accumulation)) OR TS=(Products, Lipid Accumulation)
#4	#1 AND #2 AND #3

Sup Table 4 The Cochrane library (n = 5)

Search	Query
#1	MeSH descriptor: [Polycystic Ovary Syndrome] explode all trees
#2	(Ovary Syndrome, Polycystic):ti,ab,kw OR (Syndrome, Polycystic Ovary):ti,ab,kw OR (Polycystic Ovarian Syndrome):ti,ab,kw OR (Ovarian Syndrome, Polycystic):ti,ab,kw OR (Polycystic Ovary Syndrome 1):ti,ab,kw OR (Sclerocystic Ovarian Degeneration):ti,ab,kw OR (Ovarian Degeneration, Sclerocystic):ti,ab,kw OR

Search	Query
	(Sclerocystic Ovary Syndrome):ti,ab,kw OR (Stein-Leventhal Syndrome):ti,ab,kw OR (Stein Leventhal Syndrome):ti,ab,kw OR (Syndrome, Stein-Leventhal):ti,ab,kw OR (Sclerocystic Ovaries):ti,ab,kw OR (Ovary, Sclerocystic):ti,ab,kw OR (Sclerocystic Ovary):ti,ab,kw
#3	#1 OR #2
#4	MeSH descriptor: [Metabolic Syndrome] explode all trees
#5	(Metabolic Syndromes):ti,ab,kw OR (Syndrome, Metabolic):ti,ab,kw OR (Syndromes, Metabolic):ti,ab,kw OR (Reaven Syndrome X):ti,ab,kw OR (Syndrome X, Reaven):ti,ab,kw OR (Metabolic Syndrome X):ti,ab,kw OR (Insulin Resistance Syndrome X):ti,ab,kw OR (Metabolic Cardiovascular Syndrome):ti,ab,kw OR (Cardiovascular Syndrome, Metabolic):ti,ab,kw OR (Cardiovascular Syndromes, Metabolic):ti,ab,kw OR (Syndrome, Metabolic Cardiovascular):ti,ab,kw OR (Syndrome X, Insulin Resistance):ti,ab,kw OR (Metabolic X Syndrome):ti,ab,kw OR (Syndrome, Metabolic X):ti,ab,kw OR (X Syndrome, Metabolic):ti,ab,kw OR (Syndrome X, Metabolic):ti,ab,kw OR (Dysmetabolic Syndrome X):ti,ab,kw OR (Syndrome X, Dysmetabolic):ti,ab,kw OR (Cardiometabolic Syndrome):ti,ab,kw OR (Cardiometabolic Syndromes):ti,ab,kw OR (Syndrome, Cardiometabolic):ti,ab,kw OR (Syndromes, Cardiometabolic):ti,ab,kw
#6	#4 OR #5
#7	MeSH descriptor: [Lipid Accumulation Product] explode all trees
#8	(Accumulation Product):ti,ab,kw OR (Lipid Accumulation Products):ti,ab,kw AND (Lipid Lipid Accumulation Products Product):ti,ab,kw AND (Lipid Accumulation Products):ti,ab,kw AND (Lipid Accumulation):ti,ab,kw
#9	#7 OR #8
#10	#3 AND #6 AND #9

Supplementary figure 1. The PRISMA flow diagram of the entire study selection process.

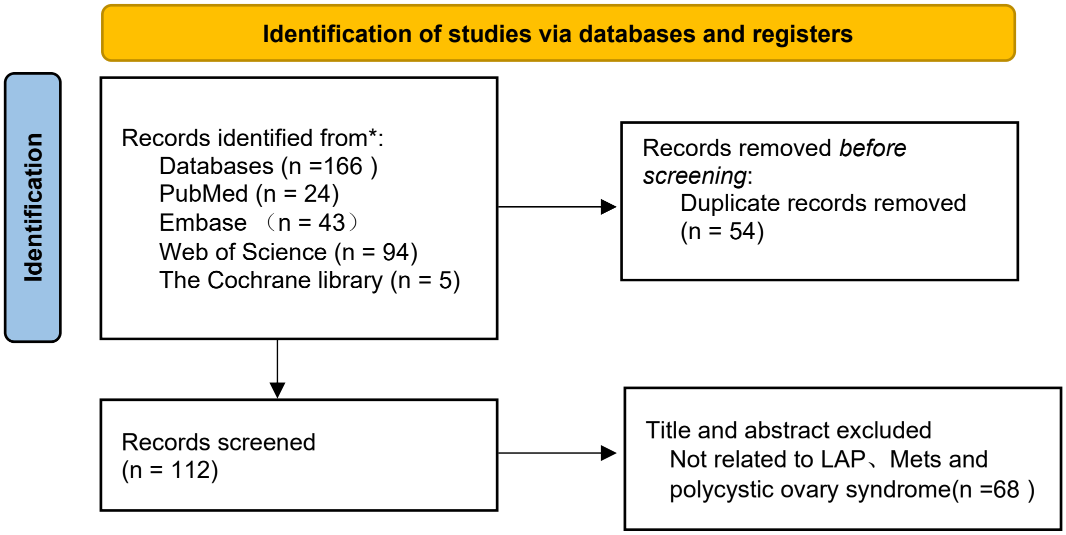

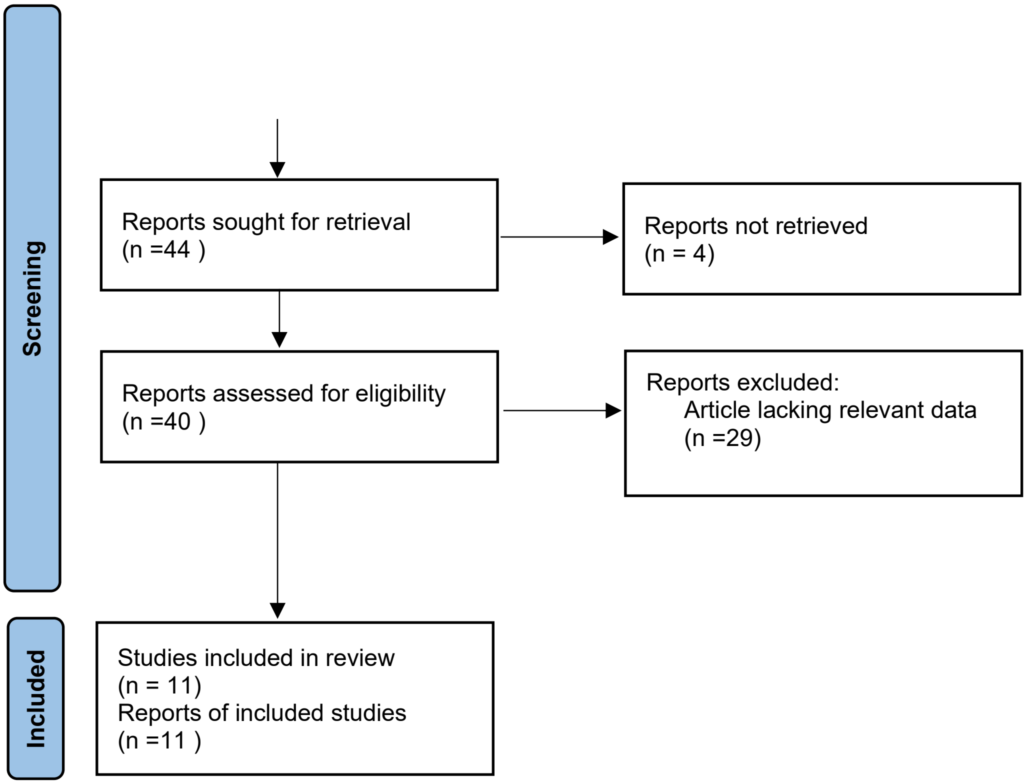
Screening
Reports sought for retrieval
(n =44)
Reports not retrieved
(n = 4)
Reports assessed for eligibility
(n =40)
Reports excluded:
Article lacking relevant data
(n =29)
Included
Studies included in review
(n = 11)
Reports of included studies
(n =11)
